# The role of estimation in Mendelian randomization: should Mendelian randomization investigations provide estimates?

**DOI:** 10.1093/ajeadv/uuaf003

**Published:** 2025-05-15

**Authors:** Benjamin Woolf, Stephen Burgess

**Affiliations:** 1 MRC Integrative Epidemiology Unit, University of Bristol, Bristol, UK; 2 School of Psychological Science, University of Bristol, Bristol, UK; 3 MRC Biostatistics Unit, University of Cambridge, Cambridge, UK; 4 Cardiovascular Epidemiology Unit, Department of Public Health and Primary Care, University of Cambridge, Cambridge, UK

## Abstract

Mendelian randomization (MR) makes causal claims by treating genetic variation in an analogous way to randomization in a clinical trial. MR investigations can be viewed as analogous to a randomized encouragement design, in that genetic variants do not determine the precise level of an exposure, but increase liability to it. As such, an MR estimate typically does not represent an achievable or well-defined causal effect in terms of the exposure, as it reflects the impact of a life-long shift in the trajectory of the exposure, which likely differs between individuals. We advocate for MR investigations to be performed to assess evidence for a causal hypothesis, rather than to estimate a well-defined causal quantity. MR estimates are useful to combine evidence across genetic variants, to assess the validity of variants as instruments, to provide confidence intervals, and to compare estimates across outcomes. However, numerical estimates from MR should not be over-interpreted. The value of an MR investigation is not to quantify the magnitude of effect for a well-defined intervention in the exposure. Instead, it provides a distinct source of evidence to increase or decrease confidence in a causal hypothesis, which can be triangulated with evidence from other sources.

## Background

Modern approaches to causal inference in epidemiology tend to emphasize the use of formal frameworks to express and estimate well-defined causal effects (1,2). A causal effect is well-defined when it is a clear and unambiguous comparison between different counterfactual states (3). Causal inference for effects which are not well-defined can be hampered by ambiguities about the nature of an exposure or intervention, its timing, and the population under investigation. If an investigator wants to estimate a precise causal quantity (the precise target of estimation is known as an “estimand”), expressing the quantity in terms of a target trial helps clarify unspoken assumptions in the analysis of data that can lead to bias if ignored (1). However, this is not the only valid paradigm for causal inference. Historical approaches to causal inference were less reliant on formalism, and tended to emphasize study design [e.g. Bradford Hill 1948 (4)] or the qualitative synthesis of diverse types of evidence [e.g. Bradford Hill 1965 (5)] to justify causal claims.

Mendelian randomization (MR) was introduced as a way of exploiting the random allocation of genetic variants to infer the causal effect of an exposure on a given health outcome using observational data (6–8). Its initial exposition effectively argued that a valid MR investigation requires each selected genetic variant to satisfy the three core instrumental variable (IV) assumptions (6), but did not use the language of instrumental variables. Later researchers formalized these assumptions using the terminology of instrumental variables (9,10): that the variant is associated with the exposure of interest, that there are no confounding pathways between the variant and outcome, and that the variant can only affect the outcome via the exposure. An association between a genetic variant which is a valid instrumental variable for the exposure of interest and the outcome implies that the exposure has a causal effect on the outcome (11–13).

Methods for the estimation of causal effects using instrumental variables, developed in the field of econometrics over the past century, have been adopted by the epidemiological community (14). Indeed, MR has been defined by some as simply “instrumental variable analysis with genetic instruments” (11). In most cases, MR investigations have provided estimates from formal instrumental variable methods (15). In occasional cases, MR investigations have simply reported on the presence and direction of association of genetic variants with the outcome, rather than providing an estimate (16,17). For example, genetic variants that modify the metabolism of carcinogens present in tobacco smoke inhalation have been used to assess the effects of smoking on bladder cancer risk (18). In this case, it would not be possible to provide a numerical estimate as the variant does not associate with any smoking behaviour, and measures of metabolism rate for these carcinogens were not available. Indeed, the trend towards estimation is symptomatic of a wider shift in MR from biologically-justified analyses to statistically-driven analyses applied without biological consideration (19,20).

We argue that MR investigations can provide useful information even though the assumptions required for the literal interpretation of causal point estimates expressed in terms of a unit change in the exposure are implausible (21). Specifically, a conventional MR analysis tests if a lifelong predisposition to greater exposure levels is associated with an increased (or decreased) risk of the outcome. As such, MR investigations can be motivated as using genetic variants to assess a causal hypothesis rather than to estimate a practically-relevant causal parameter.

**Figure 1:**
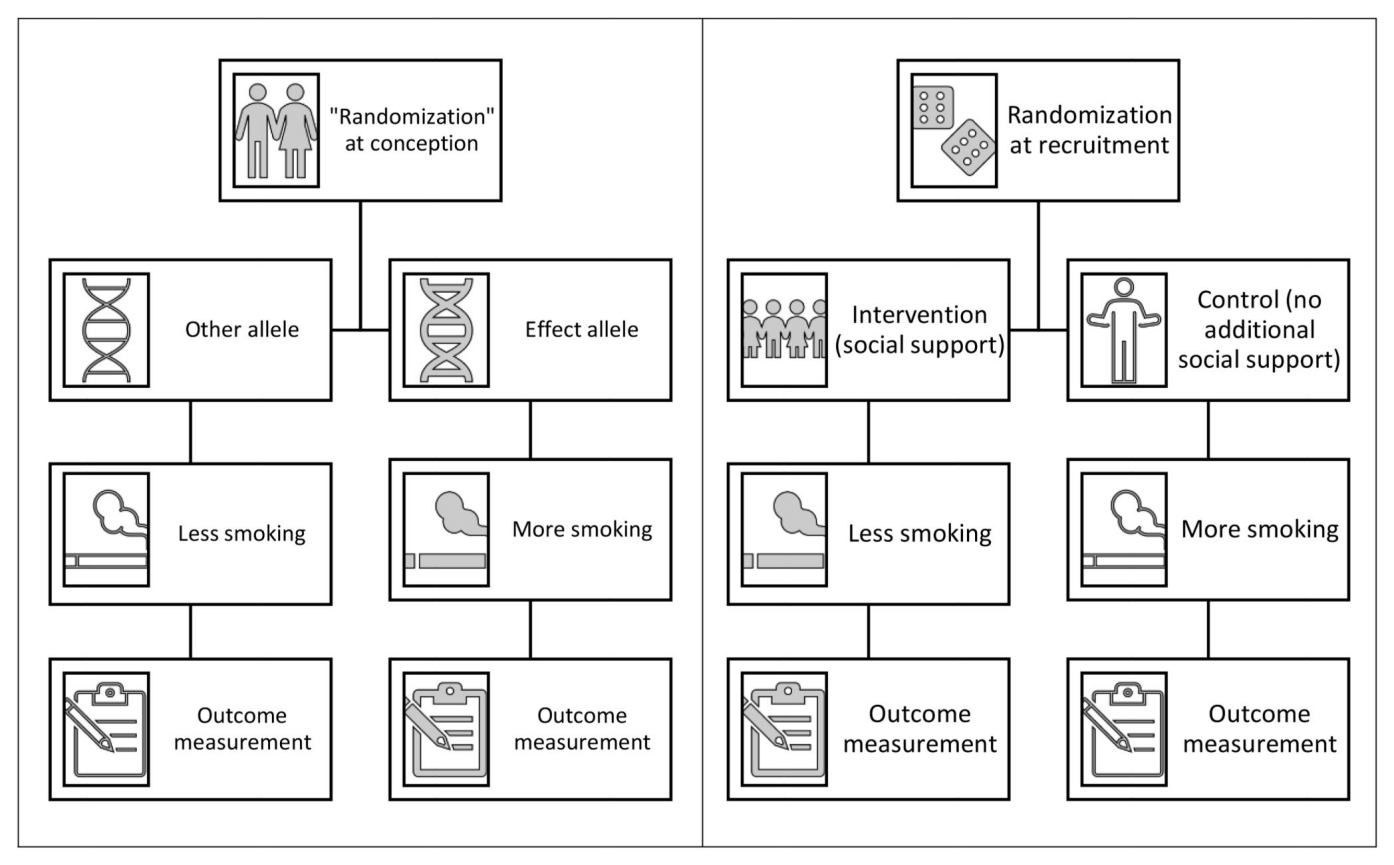
The analogy between an MR analysis for the effect of smoking and a hypothetical randomized encouragement design using social support to incentivize smoking cessation. In an encouragement design, people are not directly randomized to an exposure regimen (e.g. smoking a fixed number of cigarettes per day), but instead to some external factor (e.g. social support) which influences liability to the exposure. This is similar to an MR investigation where people are “randomized” to a genetic variant that influences levels of an exposure, rather than setting the exposure to a particular value. In both designs, the causal contrast is well-defined with respect to the intervention (i.e. social support or allele assignment). However, additional assumptions are required to interpret estimates in terms of a change in the exposure (i.e. smoking). An intention-to-treat type effect in terms of the intervention can be estimated, but a per-protocol effect in terms of the exposure cannot typically be estimated.

MR can be viewed as analogous to an encouragement design in which people are randomized to an intervention which impacts their liability to elevated or lowered levels of an exposure (Figure 1). In any randomized trial, the causal effect is well-defined with respect to allocation to the intervention. However, unless the exposure regimes in the intervention and control arms of the trial are precisely defined and strictly enforced such that they are homogeneous across individuals, the causal effect cannot be so easily interpreted as a well-defined causal effect in terms of the exposure.

Likewise, if the instrumental variable assumptions hold, the MR estimate based on a genetic variant is well defined with respect to allocation to that genetic variant, and represents the effect of changing an individual’s genotype at conception, or equivalently changing their exposure trajectory from conception in the same way as the genetic variant changes the exposure (same mechanism of intervention, same timing, and so on). But such a change likely differs from the intervention on the exposure to be implemented in practice. Using the language of Pearl’s ladder of causal inference (22), the MR estimate is defined in terms of counterfactuals (third rung), but the intervention to achieve those counterfactuals is not practical (second rung).Hence, rather than predict the quantitative impact of a specific intervention, the role of an MR estimate is to increase or decrease our qualitative confidence in an exposure as a causal risk factor for the outcome.

While we are not the first to advocate the use of MR for hypothesis testing rather than estimation (12), initial proposals suggested simply reporting the genetic association with the outcome, which is feasible with a single genetic variant, but overly simplistic when there are multiple genetic variants. Equally, there is strong advice in the epidemiological literature discouraging the reporting of results as p-values without accompanying estimates and confidence intervals (23). MR estimates represent the causal effect of a shift in lifetime liability to the exposure, although the specific magnitude and timing of the shift will likely differ between individuals (24). As such, our proposal is that MR investigations report an estimate with an appropriate measure of statistical uncertainty, but do not take an overly literal interpretation of the precise numerical estimate. We suggest that MR investigations follow existing guidelines when reporting MR estimates (23,25).

In the next section, we outline some reasons why MR estimates should not be interpreted too literally. We then discuss motivations for causal hypothesis testing. In the final section, we explore some practical implications of our arguments.

### The interpretation of Mendelian randomization estimates

#### Formal statistical assumptions for causal estimation

Two quantities that are typically targeted in instrumental variable estimation are the average causal effect, representing the population average difference in the outcome resulting from an intervention in the exposure; and the local average causal effect, representing the average difference in the outcome resulting from an intervention in the exposure among those whose value of the exposure is influenced by the instrumental variable (26). Estimation of the average causal effect requires a homogeneity assumption; a sufficient condition is that the effect of the instrument on the exposure is constant in the population (27). One challenge to the homogeneity assumption is the existence of genotype-exposure interactions (28). Estimation of the local average causal effect requires the monotonicity assumption; the effect of the instrument on the exposure is in the same direction for all individuals in the population. However, since we cannot know whose exposure was changed by the instrument, local treatment effects are applicable to an unknown subgroup and not necessarily translatable to any applied setting (29). Causal bounds can be used as an alternative to monotonicity and homogeneity (30), but these are often too imprecise to be useful in practice. Interpreting estimates also requires assuming linearity of the exposure effect on the outcome (11,31). If the MR investigation is conducted in a two-sample setting, then transportability of estimates between the two datasets is required (32). The formal statistical assumptions for causal estimation are stringent, and often implausible.

In contrast, hypothesis testing does not require these parametric assumptions (33–35). The sharp causal null hypothesis that changes in the exposure do not influence the outcome for any individual in the study population can be assessed by simply testing for an association between the genetic instrument and the outcome (or equivalently, an association between genetically-predicted levels of the exposure and the outcome) (12).

#### Contextual assumptions for causal estimation

Further to these statistical assumptions, any application of MR requires careful consideration of how the genetic variants affect the exposure, and how this compares to any proposed intervention on the exposure. To quantify the impact of an intervention, MR requires that genetic variants impact the exposure in the same way as the proposed intervention (i.e. both have quantitively and qualitatively similar effects). This is known as the gene—environment equivalence assumption (14). However, interventions typically differ from the genetic change in the exposure in a number of ways. For example, genetic effects on exposures tend to be lifelong, whereas clinical interventions are typically performed on adult patients. Thus, even when the gene—environment equivalence assumption holds, the literal interpretation of an MR estimate is the effect of an intervention which can never be exactly replicated in practice.

In contrast, to assess the mechanistic relevance of an exposure simply requires that genetic interventions are qualitatively similar to the relevant environmental intervention, which is often plausible. Hence, under a weaker version of the gene—environment equivalence assumption stating that the genetic variants affect the exposure via the same biological mechanism as the intervention, we can test whether interventions on this mechanism will affect the outcome.

#### Defining the causal risk factor

A further limitation of causal estimation is our ability to identify the true causal risk factor (12). When the specific function of a genetic variant is unclear, a causal estimate stated in terms of a particular exposure may be misleading due to misidentifying the true causal agent (36). An example is the effect of interleukin-6 receptor signalling on coronary heart disease risk (37). Measurement of the relevant causal risk factor (intracellular interleukin-6 receptor signalling) in live patients is impractical. Alternatively, the MR estimates could use a non-causal proxy of the underlying causal risk factor as the nominal exposure for the statistical analysis, such as C-reactive protein (a downstream marker of inflammation). Assuming the variants are valid instruments for interleukin-6 receptor signalling, any resulting estimates cannot be interpreted as the effect of the measured biomarker, but instead represent the effect of the unmeasured causal risk factor – in this case, interleukin-6 receptor signalling. Therefore, when presenting or interpreting an MR estimate, it is important to think carefully about the precise risk factor under investigation, and how the genetic variants affect this risk factor versus how they affect the trait that has been measured (38,39).

In contrast, we can assess a causal hypothesis expressed in terms of a broad exposure without being explicit about the precise causal risk factor when the relevant mechanism can only be ascertained crudely. For example, lifetime smoking index is a composite measure of smoking initiation, duration, heaviness, and cessation (40). Genetic variants associated with lifetime smoking index can provide evidence on the causal effect of smoking without differentiating between the precise effects of smoking initiation, duration, heaviness, or cessation. This is under the assumption that any effect of the variants acts on the outcome only via one or more of these exposures. Similarly, genetic variants associated with body mass index can provide evidence on the causal effect of adiposity without an explicit claim that the risk factor is ‘weight divided by the square of height’ (the definition of body mass index), assuming that the genetic variants influence the outcome via an adiposity-related pathway.

#### Accounting for time

MR estimates represent the causal effect of a change in the exposure, but one equivalent to changing an individual’s genetic code at conception. They therefore represent lifetime effects, whereas any intervention will be performed at a later time to the genetic ‘intervention’ and represents an effect with a limited duration at a specific time (41,42). Transporting estimates from MR analyses to clinical interventions therefore requires assumptions about how the intervention varies over time. Most exposures are not temporally constant, which can additionally bias naive MR estimates (43).

In contrast, without any further assumptions, hypothesis testing can assess the sharp causal null hypothesis – that changes in the exposure do not affect the outcome for the participants assessed in the study (33). While investigators should be careful about extrapolating the lifelong effects assessed in MR to the impact of proposed time-limited interventions, it is usually reasonable to believe that MR estimates are a reliable guide to the existence and direction of causal effects in practice.

### In defence of causal hypothesis testing

#### Causal hypothesis testing can be sufficient

Some scientific research is undertaken to obtain a precise numerical answer, particularly in the field of public health. However, much research is done to understand aetiological relationships between variables. For example, pre-clinical pharmaceutical research and Phase 2 trials aim to find evidence for target efficacy, i.e. to learn if a modifiable trait or mechanism has a potential causal effect on a disease outcome. It is only in a Phase 3 trial that the goal is to estimate the magnitude of any precisely-defined effect. Within the framework of drug development, we see Mendelian randomization as contributing evidence to the early stages on target efficacy, rather than the final phase of estimation. While further research is needed to understand how findings translate to real-world practice, there is value in assessing the impact of a shift in the distribution of an exposure on an outcome even if the shift cannot be precisely defined or replicated in practice. Increasing (or decreasing) our confidence in the causal status of a proposed mechanism is a worthwhile aim, even if the specific intervention on the mechanism is not achievable or well-defined.

Our proposal has some connections with the work of Judea Pearl, whose seminal work on structural causal models emphasizes whether there is a causal effect of an exposure on an outcome or not (44). A structural causal model, typically represented by a directed acyclic graph, encodes causal hypotheses in a non-parametric way.

#### Hypothesis testing and estimation in clinical trials

The randomized trial is often taken as a paradigm for defining and estimating the causal effect of a well-defined intervention (24). However, some influential trialists see causal hypothesis testing as an important goal of randomized trials, even for a Phase 3 trial. For example, Richard Peto and colleagues have argued that trials should be used “to distinguish reliably between the only two medically plausible alternatives: either there is no worthwhile difference in survival, or treatment confers a moderate, but worthwhile, benefit” (45). This is because trialists “are not trying to provide exact quantitative estimates of percentage risk reductions in some precisely defined population of patients. We are simply trying to determine whether or not some type of treatment – tested in a wide range of trials – produces any effect on mortality” (46). Indeed, heterogeneity between trials means that no two trials will target the exact same causal quantity, but they can provide evidence to support (or refute) the same broad causal hypothesis. While the target trial framework is a useful tool for improving the quality of observational studies, the requirement for well-defined estimation should not limit the scope of well-designed epidemiological investigations.

#### Hypothesis testing is a canonical scientific activity

The statistical essence of an MR analysis is to select genetic variants which are associated with the exposure, and test if people with elevated genetically-predicted levels of the exposure have greater risk of the outcome (8,12). This is analogous to performing an intention-to-treat analysis in a randomized trial to test if an intervention works; a statistical association between genetically-predicted levels of the exposure and the outcome provides evidence supporting a causal effect of the exposure. Epidemiology, and statistics more broadly, have moved away from null hypothesis significance testing based on the dichotomization of p-values. This is in part due to a burgeoning literature on the misinterpretation and misapplication of p-values (23). Some appear to view the abuses of p-values as justifying a rejection of hypothesis testing in general.

However, hypothesis testing pre-dates formal statistical methods, and is the primary language through which theories of the history and philosophy of science have described and analysed science (47). Within a frequentist paradigm, hypothesis testing does not require a dichotomization of results into “significant” and “non-significant” (48). Bayesian approaches can also be used – indeed the original application of Bayes’ theorem was refuting David Hume’s hypothesis about the non-existence of miracles (49). Hence, while we acknowledge that p-values can be abused, we would see a hypothesis test result (alongside an MR estimate) as a core output of an MR investigation.

#### Radical uncertainty in economics

While the techniques of instrumental variable analysis were developed in the field of econometrics, some thinkers in the wider discipline of economics believe that precise estimation of well-defined quantities should not be the aim of economic analyses. For example, John Kay and Mervyn King argue that the pervasive presence of unquantifiable uncertainty in economics means that economic models are at best an approximation of reality, and so need to be taken with “a pinch of salt” (that is, their precise numerical estimates should not be interpreted literally and uncritically) (50). They argue economists should instead perform analyses which aim to understand underlying processes and use these to inform policy. The philosophical intuition that pervasive unquantifiable uncertainty means we should not overinterpret numeric economic estimates has a prestigious lineage among early 20^th^ century economists including Benjamin Graham (the inventor of financial security analysis) (51), Friedrich Hayek (a luminary of the Austrian School) (52), Frank Knight (a founder of the Chicago School) (53), and John Maynard Keynes (the inventor of Keynesianism) (54).

The philosophy can perhaps be summarized by Keynes’ quote that he would “rather be vaguely right than precisely wrong”. This has clear implications into the trade-off between bias and variance; it is generally better to have an unbiased estimate, even if that estimate is imprecise. Additionally, if the option is to answer the precise question of interest unreliably, or a related question reliably, it would often be preferable to get a reliable answer, even if the numerical answer is not fully relevant to the precise question of interest. In the case of MR, while the intervention mimicked by the genetic variant(s) may not be similar to the proposed intervention to be applied in practice, the primary concern is whether we get reliable evidence, rather than whether we estimate the most relevant quantity.

### Implications for Mendelian randomization investigations in practice

#### Mendelian randomization estimate as a life-long causal contrast

At its most austere, an MR investigation does not attempt to estimate a causal quantity, but simply tests the sharp causal null hypothesis that the causal effect equals zero for all individuals. A serious limitation of this approach is the difficulty of interpreting a null finding, as it does not allow reporting of confidence intervals or estimating the power to detect a minimal effect of interest.

A more nuanced position (which we would typically support) is to estimate the association of genetically-predicted levels of the exposure with the outcome. Under comparatively weak assumptions, this quantity (which we call the MR estimate, and can be viewed as analogous to an intention-to-treat estimate from a clinical trial) can provide some insight into the causal effect of an exposure without having all the attributes of a well-defined causal effect.

For instance, if the genetic variant’s association with the exposure has the same direction throughout the life course (temporal monotonicity), and the direction of the causal effect of the exposure on the outcome is consistent across the life course, then the sign of the MR estimate (i.e. positive or negative) will be an accurate guide of the direction of the causal effect no matter when the genetic association is estimated. As such, MR estimates can have some meaning in predicting the effect of an intervention in the exposure even if they are not targeting an effect that is well-defined in terms of the exposure (Table 1).

**Figure 2:**
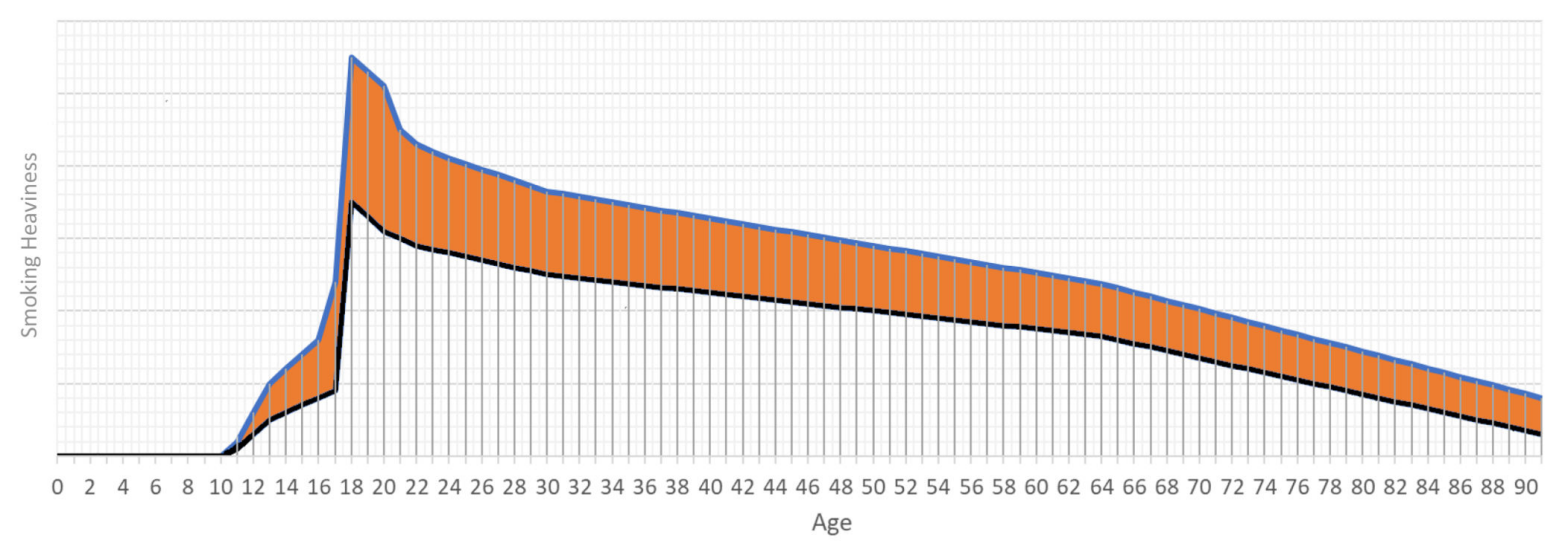
Synthetic data illustrating two hypothetical average trajectories in time for smoking heaviness among people carrying (blue line) and not carrying (black line) an additional copy of the A allele for the rs16969968 genetic variant. rs16969968 impacts on how fast people metabolize nicotine, and therefore how heavily they smoke (i.e. number of cigarette packs smoked per week) (55). If the genetic association with smoking heaviness is positive (or zero) across the life course, and the effect of smoking on the outcome is also in a consistent direction, then the MR estimate should provide a reliable indication of the direction of the causal effect no matter when the genetic association with smoking is measured.

For example, genetic variant rs16969968 in the *CHRNA5-A3-B4* gene cluster can be used to instrument smoking heaviness (i.e. number of cigarette packs smoked per week) because of its role in nicotine metabolism (55). People with the variant metabolize nicotine faster, and therefore need to smoke more to achieve the same effect, which results in greater smoking heaviness. It is not plausible for there to be a subgroup of the population where the gene’s biological function is inverted. Indeed, because the variant has a similar biological function across the life course (Figure 2), inheriting an additional copy of the A allele of rs16969968 should never associate with reduced exposure to smoking. For many outcomes, it is biologically implausible for the effect of smoking heaviness to be harmful in some time periods and protective in other time periods. As such, a positive association between rs16969968 and lung cancer risk is supportive of the directional hypothesis that an increased lifetime liability to smoking increases risk of lung cancer among people whose smoking heaviness is changed by inheriting an extra copy of the A allele of rs16969968.

Although a positive effect implies that the exposure increases risk of the outcome at some point in the life course, it may be that the effect of the exposure on the outcome is limited to a specific critical period. Similarly, we cannot make inferences about effects of the exposure that occur after the timepoint when the outcome is measured. Hence there are conditions under which an MR estimate can provide meaningful information on the direction of effect of an exposure in a particular population, although in practice a specific intervention could still be ineffective due to its timing.

#### Mendelian randomization investigations should still provide estimates

An MR estimate can be expressed in objective terms as the difference in the outcome per unit increase in genetically-predicted levels of the exposure (56). Although the causal interpretation of MR estimates in terms of a unit change in the exposure is not always clear, their calculation and presentation is often useful:

First, estimates provide important evidence on the causal relevance of the exposure. For example, a small p-value can be compatible with detecting an effect which is not of clinical relevance, while a large p-value can be compatible with low power to detect a relevant effect (57). Evaluating power can be difficult for an MR investigation. For example, if MR estimates do not represent effects which would be seen in an actual intervention, then defining the minimal relevant effect size of interest may be difficult. A practical alternative to a power calculation is the use of a positive control analysis. Detecting an association with an outcome that the exposure is believed to effect (on which the effect is similar or smaller than the hypothesized effect on the exposure) in a similar or smaller sized sample is indicative that there is sufficient power to detect the effect of interest.

Second, MR estimates can combine evidence from multiple variants to increase power to detect a true causal effect. It may be that no variant individually provides strong evidence for a causal effect of the exposure based solely on its association with the outcome, but the combination of evidence from all of the variants does. Under parametric assumptions, the causal estimate from the two-stage least squares method (or equivalently with summary-level data, the inverse-variance weighted method) represents the optimal combination of evidence from multiple instrumental variables in terms of statistical power (58). Hence, an MR estimate is a useful quantity even if it is simply viewed as a test statistic for a causal hypothesis test.

Third, MR estimates from different variants can be compared to assess violations of the instrumental variable assumptions. Heterogeneity in MR estimates using different genetic variants may indicate that the instrumental variable assumptions are violated for one or more of the variants (14,59).

Fourth, several robust methods for instrumental variable analysis have been developed, which not only provide consistent estimates under some violations of the instrumental variable assumptions, but also give close to nominal Type 1 error rates with some invalid instruments (60).

Finally, MR estimates for the same exposure may have a similar interpretation, and so can be compared, particularly if the exposure affects the outcomes in a similar way. For example, MR estimates for low-density lipoprotein cholesterol can be compared to assess the relative magnitude of impact of lipid-lowering therapy on different cardiovascular diseases (61).

#### Triangulation of evidence

The principle of triangulation is that we should approach complex questions from many different perspectives, using various approaches that make different assumptions (62). No single approach is foolproof, either in terms of internal validity (robustness to bias) or external validity (relevance to the question of interest). However, inference can be strengthened by contrasting evidence from approaches making different assumptions (and ideally with biases in different directions). Hence, evidence from an MR investigation should not be viewed in isolation. Its value is not whether it perfectly addresses a particular question about a specific intervention, but how it contributes to the evidence base about the causal status of an exposure. The strength of evidence provided will be subjective, but there is already inherent subjectivity in the validity of the instrumental variable assumptions. The primary conclusion from an MR analysis therefore should be to increase or decrease our qualitative confidence in an exposure as a causal risk factor for the outcome, not to predict the quantitative impact of a specific intervention.

## Conclusion

Some past researchers have criticized MR as being pointless (in the sense of not being worthwhile). We argue it should not be “completely pointless” (in the sense that it should not provide a point estimate), but rather it should be “somewhat pointless” (in the sense that it should provide a point estimate, but the estimate should not be interpreted as an achievable causal effect).

MR estimates provide important information on the causal nature of an exposure even if they do not quantify the expected impact of an achievable intervention. Numerical values of MR estimates should not be over-interpreted. Although MR investigations should provide an estimate, its role is to be a test statistic for the sharp causal null hypothesis, to combine evidence across multiple genetic variants, to assess the validity of variants as instruments, to provide confidence intervals, and to be compared across outcomes. A consequence is that MR estimates are generally not appropriate for use in downstream quantitative analyses, such as cost-effectiveness analyses.

The role of an MR investigation is not to provides a perfect answer to a question about the magnitude of effect for a well-defined intervention in an exposure. Instead, it provides a distinct source of evidence to support (or refute) a causal hypothesis that is robust to several sources of bias common in epidemiological studies.

## Figures and Tables

**Table 1:** Some potential goals in performing and interpreting a Mendelian randomization (MR) analysis

Goal of analysis	Interpretation of estimate
Causal estimation	For an MR estimate to have an interpretation as a well-defined causal effect, questions about who is being studied, what is the nature of the genetic “intervention”, and what is the timing and duration of the intervention must be precisely defined.
Pure hypothesis testing	Under weaker assumptions, the MR estimate has no interpretation as a causal parameter, and solely acts as a test statistic for a causal hypothesis test.
Estimate with some causal attributes and directional hypothesis testing	An MR estimate may be well-defined as an intervention in a genetic variant, but has a fuzzy interpretation as an intervention in the exposure: it has some of the attributes of a well-defined causal effect, but not all. A particular case of interest is when the sign of the estimate is a reliable guide of the direction of a causal effect of the exposure on the outcome in a given population. Sufficient conditions are temporal monotonicity (of the instrument-exposure association) and the exposure-outcome effect having a consistent direction across the life course.

## Data Availability

NA
